# A34 DEVELOPING A NOVEL MOUSE MODEL TO ELUCIDATE MECHANISMS OF ORAL TOLERANCE IN EARLY LIFE

**DOI:** 10.1093/jcag/gwad061.034

**Published:** 2024-02-14

**Authors:** R D FitzPatrick, R A Cartwright, D M Gatti, L Reynolds

**Affiliations:** University of Victoria, Victoria, BC, Canada; University of Victoria, Victoria, BC, Canada; University of Victoria, Victoria, BC, Canada; University of Victoria, Victoria, BC, Canada

## Abstract

**Background:**

Oral tolerance is the active suppression of immune responses to antigens that are first encountered in the gastrointestinal tract. When this process fails, food allergies can arise. Food allergies affect up to 6% of Canadian children underlining the need for a thorough understanding of how oral tolerance develops in childhood. Despite this, tolerance has historically been studied using *adult* rodent models instead of focusing on more physiologically relevant early-life time points.

**Aims:**

Our research aims to develop a robust mouse model to elucidate mechanisms of oral tolerance development in *early life* and we hypothesize that the mesenteric lymph nodes are a major site of food antigen-specific T cell development in early life.

**Methods:**

Prior to weaning, actively suckling mouse pups were orally gavaged with ovalbumin (OVA), an egg white component and common food allergen or water as a control. Following a series of systemic challenges with OVA, circulating antibody levels were quantified by ELISA to measure the development of oral tolerance to OVA. Additionally, splenocytes isolated from tolerized and control mice were stimulated *in vitro* with exogenous OVA and cytokines were quantified in culture supernatants via a cytometric bead array assay to assess the suppression of T cell responses to OVA in tolerized mice. One week following oral OVA exposure flow cytometry was used to characterize the expansion of T cell populations in the mesenteric lymph nodes, spleen and thymus.

**Results:**

Mice orally gavaged with OVA in early life show reduced systemic antibody production after subsequent challenges with OVA, compared to mice not orally exposed to OVA. We determined the minimum dosage of OVA required to confer tolerance in these mice and confirmed the validity of this model in both male and female C57BL/6 and BALB/c mice. Further, we detected an active suppression of IL-5 production, a cytokine indicative of a Type 2 immune response, in OVA-stimulated splenocyte cultures isolated from mice tolerized to OVA. We found a significant increase in the frequency of CD4 T cells in the mesenteric lymph nodes just 1-week following oral OVA exposure.

**Conclusions:**

We have developed a robust mouse model of oral tolerance where mice are directly exposed to OVA in an early life period while they are still actively ingesting breastmilk, and their mucosal immune system and intestinal microbiota composition are under significant development. To date, our data suggests that the gut draining mesenteric lymph nodes may be a site of food antigen-specific T cell expansion in early life.

**Funding Agencies:**

CIHRTRIANGLE

